# Transanal minimally invasive surgery for rectal cancer

**DOI:** 10.1002/ags3.12402

**Published:** 2020-10-26

**Authors:** Liam A. Devane, John P. Burke, Justin J. Kelly, Matthew R. Albert

**Affiliations:** ^1^ Department of Colorectal Surgery Beaumont Hospital Dublin Ireland; ^2^ AdventHealth Colorectal Surgery Orlando FL USA

**Keywords:** rectal cancer, TAMIS, transanal minimally invasive surgery

## Abstract

Due to the increased uptake of rectal cancer screening and the increasing rates of complete clinical response to chemoradiotherapy, more early‐stage and down‐staged rectal cancers are being treated. This has triggered surgeons to question the necessity for proctectomy and its associated morbidity and consider local excision and organ preservation in selected cases. Transanal minimally invasive surgery (TAMIS) has evolved as an oncologically safe yet cost‐effective platform for local excision of rectal tumors using traditional laparoscopic instruments. This review highlights the recent advances and current role of TAMIS in the treatment of rectal cancer.

## Graphical abstract

Increasing presentation of early‐stage and down‐staged rectal cancer has prompted organ preservation strategies in selected cases to avoid the morbidity of proctectomy. This review highlights the recent advances and current role of transanal minimally invasive surgery in the treatment of rectal cancer.

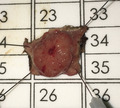

## 1 INTRODUCTION

Due to the increased uptake of rectal cancer screening and the increasing rates of complete clinical response to chemoradiotherapy, more early‐stage and down‐staged rectal cancers are being treated. While total mesorectal excision (TME) remains the gold standard of curative rectal cancer treatment, it is associated with significant morbidity and long‐term effects on anorectal, urinary, and sexual function.^1^, ^2^ Many patients require a permanent stoma and, in patients with a defunctioning stoma, 19% will not have this closed.^3^

These factors have triggered surgeons and patients to reassess the need for proctectomy and consider individualized surgical care. Thus, there is an increasing trend towards organ preservation and local excision of early rectal cancers in certain patients.^4^, ^5^ Currently, local excision is only suitable as a curative procedure in early tumors with a low risk of lymph node metastases as local lymph nodes are not adequately addressed with this technique.

The surgical platforms for local excision or rectal tumors include conventional transanal excision (TAE), colonoscopic resection, transanal endoscopic microsurgery (TEM), and transanal minimally invasive surgery (TAMIS).

Transanal excision utilizes traditional open surgery instruments under direct vision. Exposure is limited for all but the most distal rectal lesions and rates of specimen fragmentation as well as margin positivity are high.^6^

Transanal endoscopic microsurgery, first described in 1984,^7^ uses a fixed rigid resectoscope and insufflation to maintain pneumorectum and provide a stable digital or binocular magnified view. Proprietary instruments are then used to operate through the two working ports. When compared to conventional TAE, TEM provides a superior quality resection, with higher rates of negative microscopic margins, reduced rates of specimen fragmentation, and lesion recurrence but with equivalent postoperative complications.^8^ When compared to advanced colonoscopic techniques such as endoscopic mucosal resection and endoscopic submucosal dissection, TEM remains superior with respect to lesion recurrence.^9^, ^10^

However, while TEM has been used for more than 30 years, it has been slow to become incorporated into routine colorectal practice due to a steep learning curve^11^ and significant associated initial cost of the operating system.^12^ These limitations and the need for an oncologically safe and also cost‐effective procedure led to evolution of TAMIS.

Transanal minimally invasive surgery utilizes conventional laparoscopic instruments and cameras with a single incision port rather than a specialized platform. This lowers the cost of the procedure and enables the surgeon to operate with familiar instruments. Another advantage is that TAMIS obtains a 360° exposure of the rectal lumen which allows operating in multiple quadrants using the same configuration, while TEM requires repositioning of the patient or the platform. First described in 2010,^13^ TAMIS was found to be a feasible alternative to TEM, providing its benefits at a fraction of the cost without specialized instrumentation.^14^

The most common indications for TAMIS are for benign and malignant rectal tumors. However since its inception, it has been used in a wide variety of clinical scenarios from rectal prolapse and fistula repair to managing anastomotic complications and foreign body retrieval.^15^ TAMIS has also evolved to become a platform for transanal total mesorectal excision (taTME).^16^

This chapter reviews the current and future role of TAMIS in the local treatment of rectal cancer.

## 2 INDICATIONS FOR LOCAL EXCISION OF RECTAL CANCER

### 2.1 T1N0M0 rectal cancer

In Japan, the United States, and Europe,^17^, ^18^, ^19^ en bloc full‐thickness local excision is indicated for T1N0 rectal cancers with low‐risk pathologic features.

The main concern regarding local excision is the potential under‐treatment of T1 cancers that are node positive. Data from the Swedish Rectal Cancer Registry of 205 T1 rectal cancers demonstrated the overall rate of nodal metastasis was 12%^20^; however, if no adverse features were present, the rate was 6%.^20^

A meta‐analysis of 4510 patients highlighted the risk factors for nodal metastasis in the setting of T1 rectal cancer to include submucosal invasion >1 mm (odds ratio [OR]: 3.87), lymphovascular invasion (OR: 4.81), poor differentiation (OR: 5.60), and tumor budding (OR: 7.74).^21^ If any of these risk factors are present on final pathology, radical proctectomy is recommended in fit patients due to the higher risk of lymph node metastasis.

Analysis of the United States Surveillance, Epidemiology, and End Results demonstrated that local excision of T1 rectal cancer does not affect cancer‐specific survival when compared to radical surgery.^22^

### 2.2 T2N0M0 rectal cancer

The risk of positive lymph nodes in T2 cancers is approximately 22%^20^ and these patients should be recommended to undergo traditional total mesorectal excision unless enrolled in a clinical trial as local excision is still deemed somewhat experimental.^23^ However, promising results have been published for local excision of selected T2 cancers following neoadjuvant therapy.^5^, ^24^, ^25^

A randomized trial of local excision vs TME for T2N0 low rectal cancers following neoadjuvant chemoradiotherapy showed that recurrence and survival were similar between groups at over 9 years of follow‐up.^25^ The ACOSOG Z6041 study of 79 patients with T2N0 cancers treated with neoadjuvant chemoradiotherapy reported a low local recurrence rate of 4% after a median of 56 months as well as a 3 year disease‐free survival of 88%.^5^ A retrospective analysis of the National Cancer Database in the United states also showed that, for patients with T2N0 cancers, there was no difference in overall survival between radical surgery and chemoradiotherapy followed by local excision.^24^

However, for some patients, pelvic radiotherapy and/or chemotherapy can cause morbidity or functional impairment that is comparable to that associated with radical surgery.^1^ Currently, neoadjuvant radiotherapy for clinical stage T1N0 or T2N0 patients is not standard practice and can be avoided if these patients are treated with upfront curative TME.

### 2.3 Local excision after neoadjuvant chemoradiotherapy

Five year results from the GRECCAR 2 study show no difference in oncological outcomes between local excision and total mesorectal excision for selected rectal cancers that respond well to neoadjuvant chemoradiotherapy.^4^ This study included patients with T2–T3 and N0–N1 rectal cancers, ≤8 cm from the anal verge and ≤4 cm in diameter. Randomization occurred if patients had a good clinical response defined as residual tumor ≤2 cm at 8 weeks postradiotherapy. Patients undergoing local excision were planned for completion TME if the specimen pathology showed ≥T2 or R1 disease. Completion TME did not significantly affect oncologic outcomes compared with patients with ≥T2 disease treated with initial TME.

The reported pathologic complete response rate following neoadjuvant chemoradiotherapy for rectal cancer is over 20%^26^; however, this poorly correlates with clinical complete response as the majority of patients with a clinical complete response have residual cancer on pathology.^27^ If organ preservation is being pursued following a complete clinical response, there is debate whether to excise the scar or “watch and wait.” Local excision can determine if there are residual viable tumor cells giving prognostic information and selecting patients who may benefit from TME; however, local excision after chemoradiotherapy can negatively affect anorectal function.^28^

Tumor regrowth after a clinical complete response is reported as >20%^29^ and most can safely undergo salvage surgery.^30^ In the GRECCAR 2 study, tumor regrowth was only 7% after 5 years in the local excision group, albeit those with ≥T2 or R1 disease underwent completion TME.^4^ Of the 81 patients treated with local excision in the pre‐treatment analysis, 38 (47%) had indications for completion TME with 28 (35%) undergoing TME. The high rate of organ preservation and excellent oncologic results in this study are promising as the GRECCAR 2 cohort included patients with a good response and not just a complete clinical response.

### 2.4 Advanced disease

Local excision with TAMIS may be considered for more advanced disease as a less invasive but oncologically inferior alternative to radical excision in patients with prohibitive comorbidities or who refuse radical surgery. Non‐surgical treatment options such as palliative chemoradiotherapy should be considered,^31^ especially for >T2 disease due to the high risk of local recurrence and symptoms. In the palliative setting, treatment plans should be tailored to consider patient symptoms, comorbidities, expected survival, and tumor stage.

### 2.5 Preoperative staging

A full physical examination, including rigid proctoscopy and digital rectal exam should be performed by the operating surgeon to determine the distance of the lesion from the anal verge in centimeters, mobility, size, percentage of circumference involvement, anterior or posterior location, its position in relation to the sphincter complex and to the valves of Houston. As with any rectal lesion, all patients should undergo a full colonoscopy to rule out any synchronous pathology. While this text discusses TAMIS for rectal cancer, approximately 18.8% of transanally resected clinically benign adenoma will have invasive adenocarcinoma on their final pathology.^32^ Despite this, radiologic evaluation of clinically benign lesions may not be necessary.^33^

Preoperative radiologic evaluation aids identification of lesions suitable for local excision by determining the radiological tumor, node, and metastasis stage. Systemic staging requires a computed tomography (CT) of the thorax, abdomen, and pelvis. Local staging modalities are magnetic resonance imaging (MRI) and endorectal ultrasound (ERUS). When trying to distinguish between early lesions (T1 and T2 stage), the resolution of ERUS is better, with over‐staging of T1 tumors observed in 11% of cases compared to 100% of those staged with MRI.^34^ The accurate detection of involved lymph nodes remains challenging, but MRI appears superior in this setting in addition to assessment of the circumferential resection margin. Thus, ERUS and MRI should be considered complementary in assessment of early rectal cancer. Biopsy or local excision prior to radiological evaluation may lead to reactive lymphadenopathy and, consequently, unnecessary over‐staging.

### 2.6 Tamis technique

**FIGURE 1:**
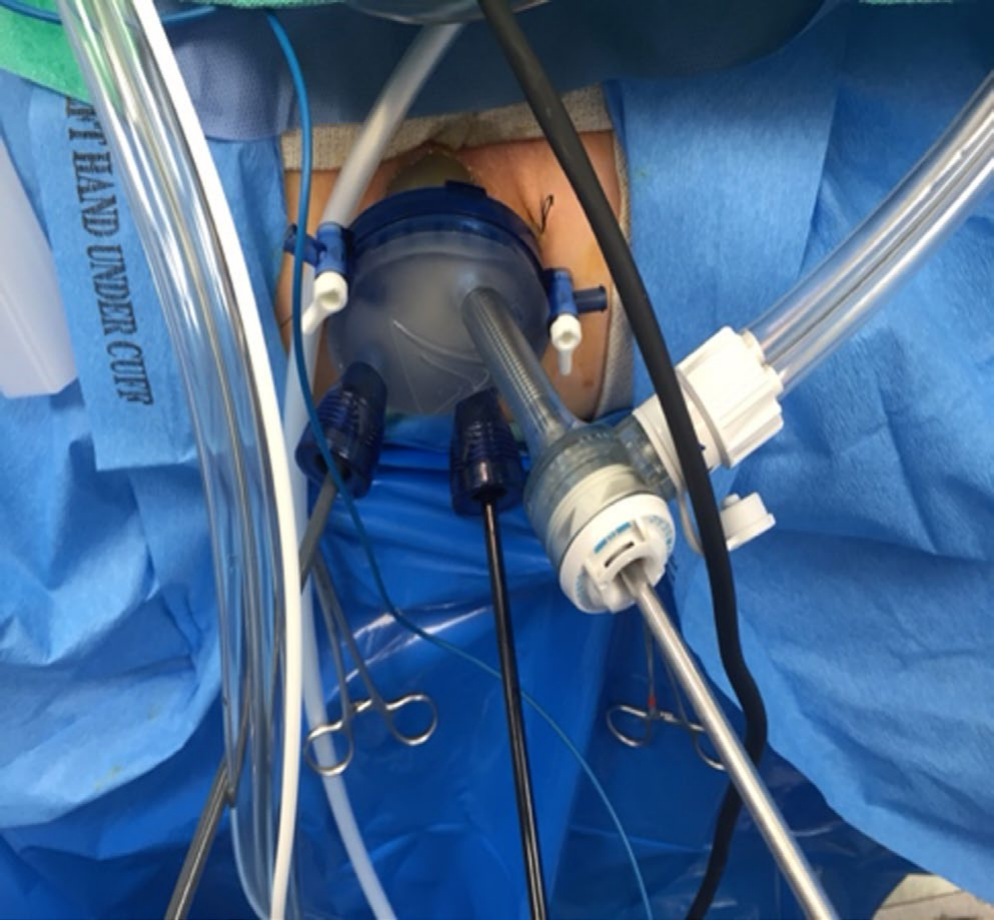
GelPOINT path transanal minimally invasive surgery port with three trocars. The camera is in the 12 o'clock Airseal™ port held by the assistant. The bottom two ports contain instruments used by the operator

Mechanical bowel preparation should be administered preoperatively, and prophylactic antibiotics given. Following informed consent, patients should be administered general anesthesia; however, spinal anesthesia may be considered in high‐risk patients.^35^ Positioning in the high dorsal lithotomy allows access to lesions in any location. Following antiseptic skin preparation and draping, the transanal port is inserted and sutured in place (Figure 1).

Pneumorectum is established using CO_2_ insufflation with a flow rate of 40 mm Hg/min and a pressure of 15 mm Hg. The Airseal insufflator (Airseal™, Surgiquest) can improve pneumorectum stability at lower pressures, even during use of suction, in addition to dramatically reducing intraluminal smoke.^36^ A 5 mm, 30° or 45° camera provides optimal visualization and working space in the tight confines of the rectum compared with 10 mm or 0° cameras. Image stabilization and sufficient visualization of the working space is dependent on an experienced assistant surgeon.

**FIGURE 2:**
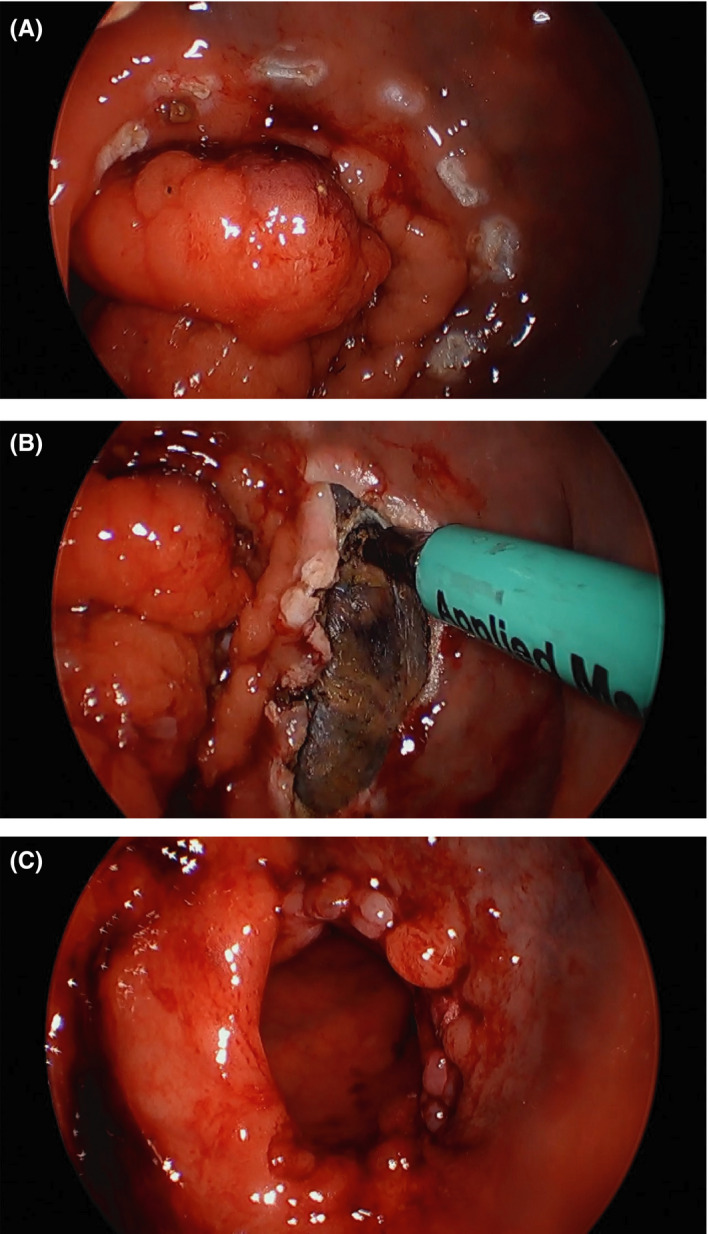
A, Electrocautery marking of margin around tumor; B, Full‐thickness excision of tumor; C, Suture closure of defect

A premium should be placed on the surgical technique and quality of resection; a non‐fragmented specimen with negative margins has repeatedly demonstrated the lowest risk of recurrence. A 10 mm margin is first scored out on the mucosa using electrocautery to maintain orientation and mark the resection (Figure 2A). Standard monopolar electrocautery with a spatula, pinpoint, or L‐hook cautery allows precise dissection of the tumor and is cheap and reusable. Energy devices can also be used with the principal advantage of hemostasis, albeit with increased costs. Most practitioners utilize standard, straight non‐articulating laparoscopic instruments. Handling of the tumor or polyp with graspers should be avoided and reduced to the surrounding mucosa to limit specimen fragmentation. For malignant lesions, a full‐thickness excision must be performed with the objective of obtaining a 1 cm minimum negative margin. Stage I rectal cancers rarely have intramural or mesenteric spread (2.7%) and if this occurs the maximum extent is 4 mm.^37^ In contrast to the historical description of a simple full‐thickness incision into perirectal fat, we support a pyramidal, volumetric excision containing an adequate specimen of perirectal fat (Figure 2B). This assures an adequate resection with negative margins, in addition to potentially retrieving surrounding lymph nodes for pathologic sampling. Aggressive mesorectal excision should not breach the mesorectal fascia as it will make future total mesorectal excision challenging, if it is required.

**FIGURE 3:**
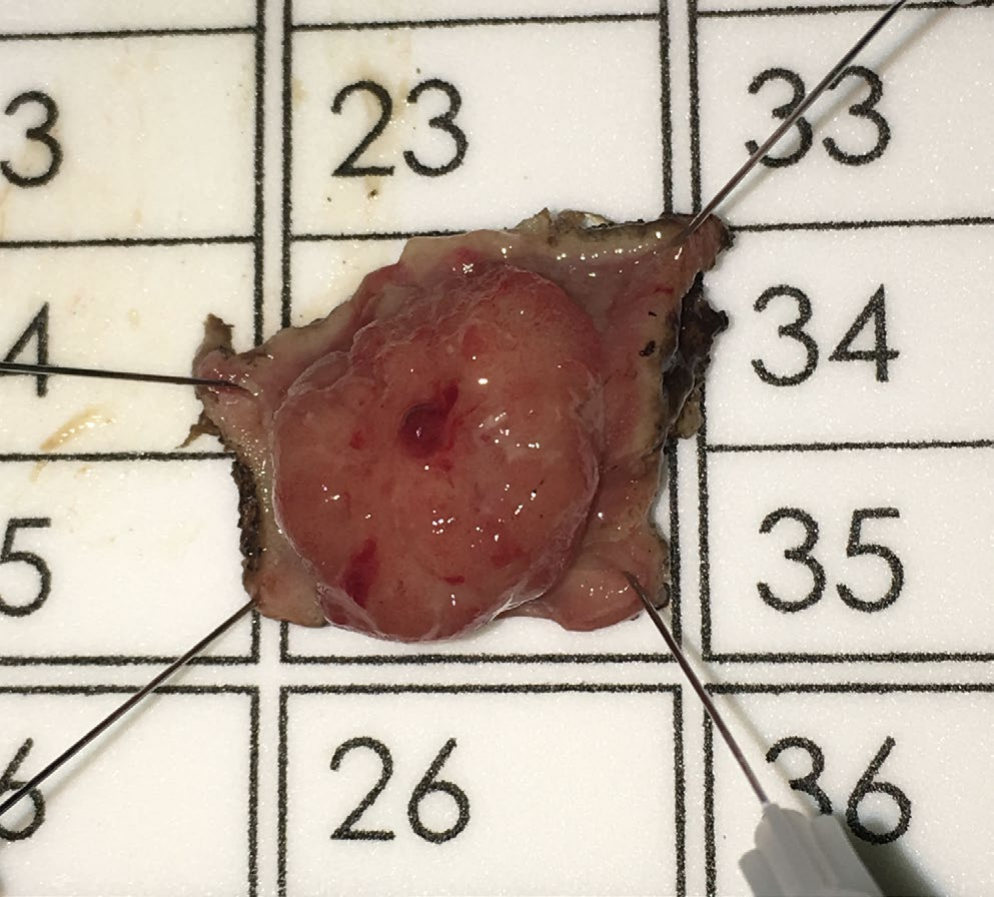
Specimen pinned for histologic assessment

Specimen extraction should be performed at completion of resection and prior to defect closure to maintain specimen integrity and avoid accidental proximal migration. The majority of platforms accommodate this by allowing removal of the faceplate; however, some ports require removal of the entire device with reinsertion for closure. Irrigation of the excision bed with dilute betadine or chlorhexidine, presumably for its tumoricidal and bactericidal effects, is a common practice; however, no evidence‐based literature exists to support this. The specimen should be pinned and orientated to ensure accurate pathologic assessment (Figure 3).

Rectal wall or mucosal defects are closed in a full‐thickness manner with absorbable suture material (Figure 2C). The use of a V‐Loc™ suture (Covidien) can facilitate continuous closure by maintaining tension and negating the need for knot‐tying. Conversely, closure can be performed in an interrupted fashion with knot‐tying facilitated by disposable devices such as the Cor‐Knot® System (LSI Solutions) or by laparoscopic knot pushers. The use of modern suturing devices can significantly shorten the learning curve at the expense of increased procedural costs. Specialized silver beads with applicators initially designed for use with the TEM system are frequently used; however, several other simpler laparoscopic knot‐tying devices and methods have since become available. Whether the defect should be closed or left open remains an issue of debate. Surgeons advocating closure, site expedited healing, less bleeding, and diminished risk of stricture. Conversely, other practitioners believe it to be solely aesthetic, increasing operative time without any improvement in healing. A series of 75 patients with 1 year of follow‐up after undergoing TAMIS resection has demonstrated no difference between groups.^38^

## 3 OPERATIVE OUTCOMES

### 3.1 Complications

The complications attributable to TAMIS reported in the literature to date have been minor, the commonest being post‐procedural hemorrhage, which can occur early postoperatively or be delayed in presentation.^39^, ^40^, ^41^, ^42^ Cases of post‐procedural hemorrhage that do not stop spontaneously have in all cases been managed successfully either endoscopically or with examination under anesthesia and over‐sewing. Scrotal emphysema appears to resolve spontaneously.^39^ Transient pyrexia responds to oral antibiotic treatment^40^ and urinary retention resolved following urethral catheterization.^41^

Serious consideration must be given to the risk of inadvertent or unplanned intraperitoneal entry and its recognition is imperative.^39^ This is more likely to happen during the resection of larger lesions, with an anterior location, with an uppermost level over 10 cm from the anal verge.^42^ The largest TAMIS series published to date report a 2% incidence of peritoneal entry.^39^ The management of this complication must be individualized depending on the size of the defect and the patient. These defects may be repaired either transanally or with a combined abdominal approach. The decision to fashion a subsequent diverting ostomy must also be individualized to the patient and their ability to tolerate a subsequent anastomotic leak with reported incidence of diverting ostomy in the setting of TEM being 0%–14%.^42^, ^43^

### 3.2 Oncologic quality

Being a relatively new and novel surgical modality, there is limited short‐term oncological follow‐up for TAMIS excisions. These were discussed in a recent systematic review that included 259 patients and specifically addressed the rates of recurrence of benign and malignant rectal neoplasms after TAMIS surgery.^44^ Altogether, there were 2.7% incidence of recurrence reported with 7.1‐month mean follow‐up. A review of TAMIS outcomes demonstrates that more than 60% of surgeons consistently performed full‐thickness excision while 10% performed only submucosal excisions. Thirty percent of surgeons performed both full thickness excisions and submucosal excisions depending on the location of tumor and pathology.

### 3.3 Functional/anorectal physiology

A recent series of 25 patients undergoing TAMIS with a SILS™ port assessed at 3 months following surgery with a combination of endoanal ultrasonography and a fecal incontinence severity index (FISI) score did not show anal sphincter injury or fecal incontinence‐related symptoms, respectively.^35^ A further series of 37 patients undergoing TAMIS revealed an improvement in FISI score for patients with impaired preoperative continence.^45^ Conversely, analysis of anorectal function following TEM has shown at 3 months following excision, the mean Wexner continence score deteriorates, with associated symptoms of fecal urgency, but returns to baseline within 5 years.^46^ Post‐operative manometry values at 3 months are significantly lower than at baseline but return to preoperative values at 1 year.^46^, ^47^ Thus, there has been no demonstration to date that the performance of TAMIS adversely affects patient continence.

## 4 FOLLOW‐UP

Pathologic analysis should take place at a multidisciplinary team meeting and, for early T1 cancers, national guidelines should be followed. The National Comprehensive Cancer Network recommend a history, physical examination, rigid proctoscopy, and serum CEA level should be performed every 3 months for 2 years, then every 6 months for a total of 5 years. A full colonoscopy at 1 and 3 years following resection should be performed and every 5 years thereafter to identify metachronous lesions. It is the authors' practice to further perform an MRI of the pelvis to identify suspicious lymph nodes every 6 months for the first 2 years following resection of early T1 cancers.

If the tumor is a high‐risk T1 or more advanced lesion, a discussion may take place regarding completion TME unless the patient is enrolled in a clinical trial or there are other factors precluding them from further surgery.

## 5 FUTURE DIRECTION

A number of ongoing trials are assessing the outcomes of local excision of rectal cancer in combination with chemoradiotherapy and as such the role of TAMIS in rectal cancer treatment may expand.

Following local excision in high‐risk T1 and low‐risk T2 rectal cancers, the outcome of completion TME vs adjuvant chemoradiotherapy is under investigation in the TESAR Dutch trial.^48^ The GRECCAR12 study is investigating the role of neoadjuvant FOLFIRINOX in addition to radiochemotherapy for T2/T3 N0/N1 rectal cancer to determine if this can increase the rate of organ preservation. All good responders will undergo local excision to histologically assess if TME is indicated.^49^ STAR‐TREK is designed as a three‐arm trial of TME vs watch and wait vs local excision for T1–T3b N0 rectal cancer following either long‐course chemoradiotherapy or short‐course radiotherapy.^50^

Advances in TAMIS technique may be possible with robotic platforms which have the potential to improve ergonomics in the confined space of a transanal approach. Early reports have shown robotic‐TAMIS to have similar short‐term outcomes but at a higher cost.^51^ Other innovations which may improve this technique are real‐time intraoperative assessment of tissue and margins with mass spectrometry^52^ or immunofluorescence.^53^

## 6 CONCLUSIONS

Based on current evidence, TAMIS, in experienced hands, results in the high‐quality local excision of early rectal cancers with low rates of margin positivity and low recurrence rates. TAMIS has an excellent morbidity profile with no long‐term adverse effect on continence.

The role of TAMIS in treating more advanced rectal cancer remains the subject of ongoing trials. As such, the authors recommend that for tumors that are high‐risk T1 or more advanced, a discussion should take place regarding completion TME. TAMIS can also be considered as a palliative procedure to patients with metastatic disease, which would potentially avoid complications of a major surgery.

Transanal minimally invasive surgery has enabled the performance of high‐quality local excision of rectal lesions by many colorectal surgeons, integrating transanal endoscopic surgery into mainstream practice. As with all new surgical techniques, appropriate training must be ensured, and the continued assessment and assurance of oncological outcome must be maintained.

## DISCLOSURE

Funding: There was no funding provided for this submission.

Conflict of interests: Author MA is a consultant for, and receives honorarium for education/courses from, Applied Medical, Stryker Endoscopy, and Conmed. MA is a consultant and advisory board member for Proximie. MA is a consultant and receives financial support for research from Astellas. MA is a consultant for Cooper Surgical.

Author contribution: Liam Devane performed initial manuscript, editing and final manuscript. John Burke and Justin Kelly performed initial manuscript structure, editing, and final proofing. Matthew Albert performed editing, illustrations/photographs and final proofing.

## References

[1] Stephens RJ , Thompson LC , Quirke P , Steele R , Grieve R , Couture J , et al. Impact of short‐course preoperative radiotherapy for rectal cancer on patients' quality of life: data from the Medical Research Council CR07/National Cancer Institute of Canada Clinical Trials Group C016 randomized clinical trial. J Clin Oncol. 2010;28(27):4233–9. 20585099 10.1200/JCO.2009.26.5264

[2] Peeters K , van de Velde C , Leer J , Martijn H , Junggeburt J , Kranenbarg EK , et al. Late side effects of short‐course preoperative radiotherapy combined with total mesorectal excision for rectal cancer: increased bowel dysfunction in irradiated patients–a Dutch colorectal cancer group study. J Clin Oncol. 2005;23(25):6199–206. 16135487 10.1200/JCO.2005.14.779

[3] Zhou X , Wang B , Li F , Wang J , Fu W . Risk factors associated with nonclosure of defunctioning stomas after sphincter‐preserving low anterior resection of rectal cancer: a meta‐analysis. Dis Colon Rectum. 2017;60(5):544–54. 28383455 10.1097/DCR.0000000000000819

[4] Rullier E , Vendrely V , Asselineau J , Rouanet P , Tuech J‐J , Valverde A , et al. Organ preservation with chemoradiotherapy plus local excision for rectal cancer: 5‐year results of the GRECCAR 2 randomised trial. Lancet Gastroenterol Hepatol. 2020;5(5):465–74. 32043980 10.1016/S2468-1253(19)30410-8

[5] Garcia‐Aguilar J , Renfro LA , Chow OS , Shi Q , Carrero XW , Lynn PB , et al. Organ preservation for clinical T2N0 distal rectal cancer using neoadjuvant chemoradiotherapy and local excision (ACOSOG Z6041): results of an open‐label, single‐arm, multi‐institutional, phase 2 trial. Lancet Oncol. 2015;16(15):1537–46. 26474521 10.1016/S1470-2045(15)00215-6 PMC4984260

[6] Paty PB , Nash GM , Baron P , Zakowski M , Minsky BD , Blumberg D , et al. Long‐term results of local excision for rectal cancer. Ann Surg. 2002;236(4):522–9; discussion 9–30. 12368681 10.1097/00000658-200210000-00015 PMC1422607

[7] Buess G , Hutterer F , Theiss J , Bobel M , Isselhard W , Pichlmaier H . A system for a transanal endoscopic rectum operation. Chirurg. 1984;55(10):677–80. 6510078

[8] Clancy C , Burke JP , Albert MR , O'Connell PR , Winter DC . Transanal endoscopic microsurgery versus standard transanal excision for the removal of rectal neoplasms: a systematic review and meta‐analysis. Dis Colon Rectum. 2015;58(2):254–61. 25585086 10.1097/DCR.0000000000000309

[9] Barendse R , van den Broek F , Dekker E , Bemelman W , de Graaf E , Fockens P , et al. Systematic review of endoscopic mucosal resection versus transanal endoscopic microsurgery for large rectal adenomas. Endoscopy. 2011;43(11):941–9. 21971923 10.1055/s-0030-1256765

[10] Arezzo A , Passera R , Saito Y , Sakamoto T , Kobayashi N , Sakamoto N , et al. Systematic review and meta‐analysis of endoscopic submucosal dissection versus transanal endoscopic microsurgery for large noninvasive rectal lesions. Surg Endosc. 2014;28(2):427–38. 24149849 10.1007/s00464-013-3238-3

[11] Barendse RM , Dijkgraaf MG , Rolf UR , Bijnen AB , Consten ECJ , Hoff C , et al. Colorectal surgeons' learning curve of transanal endoscopic microsurgery. Surg Endosc. 2013;27(10):3591–602. 23572216 10.1007/s00464-013-2931-6

[12] Maslekar S , Pillinger SH , Sharma A , Taylor A , Monson JR . Cost analysis of transanal endoscopic microsurgery for rectal tumours. Colorectal Dis. 2007;9(3):229–34. 17298620 10.1111/j.1463-1318.2006.01132.x

[13] Atallah S , Albert M , Larach S . Transanal minimally invasive surgery: a giant leap forward. Surg Endosc. 2010;24(9):2200–5. 20174935 10.1007/s00464-010-0927-z

[14] Melin AA , Kalaskar S , Taylor L , Thompson JS , Ternent C , Langenfeld SJ . Transanal endoscopic microsurgery and transanal minimally invasive surgery: is one technique superior? Am J Surg. 2016;212(6):1063–7. 27810138 10.1016/j.amjsurg.2016.08.017

[15] Keller DS . Applications beyond local excision In: Atallah S, editor. Transanal minimally invasive surgery (TAMIS) and transanal total mesorectal excision (taTME). Cham: Springer International Publishing, 2019; p. 143–52.

[16] Atallah S , Albert M , deBeche‐Adams T , Nassif G , Polavarapu H , Larach S . Transanal minimally invasive surgery for total mesorectal excision (TAMIS–TME): a stepwise description of the surgical technique with video demonstration. Tech Coloproctol. 2013;17(3):321–5. 23377536 10.1007/s10151-012-0971-x

[17] Hashiguchi Y , Muro K , Saito Y , Ito Y , Ajioka Y , Hamaguchi T , et al. Japanese Society for Cancer of the Colon and Rectum (JSCCR) guidelines 2019 for the treatment of colorectal cancer. Int J Clin Oncol. 2020;25(1):1–42. 31203527 10.1007/s10147-019-01485-z PMC6946738

[18] Monson JRT , Weiser MR , Buie WD , Chang GJ , Rafferty JF , Buie WD , et al. Practice parameters for the management of rectal cancer (revised). Dis Colon Rectum. 2013;56(5):535–50. 23575392 10.1097/DCR.0b013e31828cb66c

[19] Glynne‐Jones R , Wyrwicz L , Tiret E , Brown G , Rödel C , Cervantes A , et al. Rectal cancer: ESMO Clinical Practice Guidelines for diagnosis, treatment and follow‐up. Ann Oncol. 2018;29(Suppl 4):iv263. 29741565 10.1093/annonc/mdy161

[20] Saraste D , Gunnarsson U , Janson M . Predicting lymph node metastases in early rectal cancer. Eur J Cancer. 2013;49(5):1104–8. 23122785 10.1016/j.ejca.2012.10.005

[21] Beaton C , Twine CP , Williams GL , Radcliffe AG . Systematic review and meta‐analysis of histopathological factors influencing the risk of lymph node metastasis in early colorectal cancer. Colorectal Dis. 2013;15(7):788–97. 23331927 10.1111/codi.12129

[22] Bhangu A , Brown G , Nicholls RJ , Wong J , Darzi A , Tekkis P . Survival outcome of local excision versus radical resection of colon or rectal carcinoma: a Surveillance, Epidemiology, and End Results (SEER) population‐based study. Ann Surg. 2013;258(4):563–9; discussion 9–71. 23979270 10.1097/SLA.0b013e3182a4e85a

[23] Borstlap WAA , van Oostendorp SE , Klaver CEL , Hahnloser D , Cunningham C , Rullier E , et al. Organ preservation in rectal cancer: a synopsis of current guidelines. Colorectal Dis. 2018;20(3):201–10. 10.1111/codi.13960 29136328

[24] Lee L , Kelly J , Nassif GJ , Atallah SB , Albert MR , Shridhar R , et al. Chemoradiation and local excision for T2N0 rectal cancer offers equivalent overall survival compared to standard resection: a national cancer database analysis. J Gastrointest Surg. 2017;21(10):1666–74. 28819913 10.1007/s11605-017-3536-5

[25] Lezoche E , Baldarelli M , Lezoche G , Paganini AM , Gesuita R , Guerrieri M . Randomized clinical trial of endoluminal locoregional resection versus laparoscopic total mesorectal excision for T2 rectal cancer after neoadjuvant therapy. Br J Surg. 2012;99(9):1211–8. 22864880 10.1002/bjs.8821

[26] Al‐Sukhni E , Attwood K , Mattson DM , Gabriel E , Nurkin SJ . Predictors of pathologic complete response following neoadjuvant chemoradiotherapy for rectal cancer. Ann Surg Oncol. 2016;23(4):1177–86. 26668083 10.1245/s10434-015-5017-y PMC5295136

[27] Hiotis SP , Weber SM , Cohen AM , Minsky BD , Paty PB , Guillem JG , et al. Assessing the predictive value of clinical complete response to neoadjuvant therapy for rectal cancer: an analysis of 488 patients. J Am Coll Surg. 2002;194(2):131–5; discussion 5–6. 11848629 10.1016/s1072-7515(01)01159-0

[28] Habr‐Gama A , Lynn PB , Jorge JMN , São Julião GP , Proscurshim I , Gama‐Rodrigues J , et al. Impact of organ‐preserving strategies on anorectal function in patients with distal rectal cancer following neoadjuvant chemoradiation. Dis Colon Rectum. 2016;59(4):264–9. 26953984 10.1097/DCR.0000000000000543

[29] Chadi SA , Malcomson L , Ensor J , Riley RD , Vaccaro CA , Rossi GL , et al. Factors affecting local regrowth after watch and wait for patients with a clinical complete response following chemoradiotherapy in rectal cancer (InterCoRe consortium): an individual participant data meta‐analysis. Lancet Gastroenterol Hepatol. 2018;3(12):825–36. 30318451 10.1016/S2468-1253(18)30301-7

[30] Dattani M , Heald RJ , Goussous G , Broadhurst J , São Julião GP , Habr‐Gama A , et al. Oncological and survival outcomes in watch and wait patients with a clinical complete response after neoadjuvant chemoradiotherapy for rectal cancer: a systematic review and pooled analysis. Ann Surg. 2018;268(6):955–67. 29746338 10.1097/SLA.0000000000002761

[31] Tyc‐Szczepaniak D , Wyrwicz L , Kepka L , Michalski W , Olszyna‐Serementa M , Palucki J , et al. Palliative radiotherapy and chemotherapy instead of surgery in symptomatic rectal cancer with synchronous unresectable metastases: a phase II study. Ann Oncol. 2013;24(11):2829–34. 24013512 10.1093/annonc/mdt363

[32] Serra‐Aracil X , Caro‐Tarrago A , Mora‐Lopez L , Casalots A , Rebasa P , Navarro‐Soto S . Transanal endoscopic surgery with total wall excision is required with rectal adenomas due to the high frequency of adenocarcinoma. Dis Colon Rectum. 2014;57(7):823–9. 24901682 10.1097/DCR.0000000000000139

[33] Lee L , Arbel L , Albert MR , Atallah SB , Hill J , Monson JRT . Radiologic evaluation of clinically benign rectal neoplasms may not be necessary before local excision. Dis Colon Rectum. 2018;61(10):1163–9. 30113341 10.1097/DCR.0000000000001168

[34] Fernández‐Esparrach G , Ayuso‐Colella JR , Sendino O , Pagés M , Cuatrecasas M , Pellisé M , et al. EUS and magnetic resonance imaging in the staging of rectal cancer: a prospective and comparative study. Gastrointest Endosc. 2011;74(2):347–54. 21802588 10.1016/j.gie.2011.03.1257

[35] Lee TG , Lee SJ . Transanal single‐port microsurgery for rectal tumors: minimal invasive surgery under spinal anesthesia. Surg Endosc. 2014;28(1):271–80. 24061623 10.1007/s00464-013-3184-0

[36] Bislenghi G , Wolthuis AM , de Buck van Overstraeten A , D'Hoore A . AirSeal system insufflator to maintain a stable pneumorectum during TAMIS. Tech Coloproctol. 2015;19(1):43–5. 25421704 10.1007/s10151-014-1244-7

[37] Shimada Y , Takii Y , Maruyama S , Ohta T . Intramural and mesorectal distal spread detected by whole‐mount sections in the determination of optimal distal resection margin in patients undergoing surgery for rectosigmoid or rectal cancer without preoperative therapy. Dis Colon Rectum. 2011;54(12):1510–20. 22067179 10.1097/DCR.0b013e318233fc4a

[38] Hahnloser D , Cantero R , Salgado G , Dindo D , Rega D , Delrio P . Transanal minimal invasive surgery for rectal lesions: should the defect be closed? Colorectal Dis. 2015;17(5):397–402. 25512176 10.1111/codi.12866

[39] Albert MR , Atallah SB , deBeche‐Adams TC , Izfar S , Larach SW . Transanal minimally invasive surgery (TAMIS) for local excision of benign neoplasms and early‐stage rectal cancer: efficacy and outcomes in the first 50 patients. Dis Colon Rectum. 2013;56(3):301–7. 23392143 10.1097/DCR.0b013e31827ca313

[40] Gorgun IE , Aytac E , Costedio MM , Erem HH , Valente MA , Stocchi L . Transanal endoscopic surgery using a single access port: a practical tool in the surgeon's toybox. Surg Endosc. 2014;28(3):1034–8. 24178864 10.1007/s00464-013-3267-y

[41] Hompes R , Ris F , Cunningham C , Mortensen NJ , Cahill RA . Transanal glove port is a safe and cost‐effective alternative for transanal endoscopic microsurgery. Br J Surg. 2012;99(10):1429–35. 22961525 10.1002/bjs.8865

[42] Marks JH , Frenkel JL , Greenleaf CE , D'Andrea AP . Transanal endoscopic microsurgery with entrance into the peritoneal cavity: is it safe? Dis Colon Rectum. 2014;57(10):1176–82. 25203373 10.1097/DCR.0000000000000208

[43] Eyvazzadeh DJ , Lee JT , Madoff RD , Mellgren AF , Finne CO . Outcomes after transanal endoscopic microsurgery with intraperitoneal anastomosis. Dis Colon Rectum. 2014;57(4):438–41. 24608299 10.1097/DCR.0000000000000063

[44] Martin‐Perez B , Andrade‐Ribeiro GD , Hunter L , Atallah S . A systematic review of transanal minimally invasive surgery (TAMIS) from 2010 to 2013. Tech Coloproctol. 2014;18(9):775–88. 24848524 10.1007/s10151-014-1148-6

[45] Schiphorst AH , Langenhoff BS , Maring J , Pronk A , Zimmerman DD . Transanal minimally invasive surgery: initial experience and short‐term functional results. Dis Colon Rectum. 2014;57(8):927–32. 25003287 10.1097/DCR.0000000000000170

[46] Allaix ME , Rebecchi F , Giaccone C , Mistrangelo M , Morino M . Long‐term functional results and quality of life after transanal endoscopic microsurgery. Br J Surg. 2011;98(11):1635–43. 21713758 10.1002/bjs.7584

[47] Kreis ME , Jehle EC , Haug V , Manncke K , Buess GF , Becker HD , et al. Functional results after transanal endoscopic microsurgery. Dis Colon Rectum. 1996;39(10):1116–21. 8831526 10.1007/BF02081411

[48] Borstlap WAA , Tanis PJ , Koedam TWA , Marijnen CAM , Cunningham C , Dekker E , et al. A multi‐centred randomised trial of radical surgery versus adjuvant chemoradiotherapy after local excision for early rectal cancer. BMC Cancer. 2016;16:513. 27439975 10.1186/s12885-016-2557-x PMC4955121

[49] Optimisation of Response for Organ Preservation in Rectal Cancer: Neoadjuvant Chemotherapy and Radiochemotherapy vs. Radiochemotherapy. [cited Aug 3, 2015]. Available from https://ClinicalTrials.gov/show/NCT02514278

[50] Rombouts AJM , Al‐Najami I , Abbott NL , Appelt A , Baatrup G , Bach S , et al. Can we Save the rectum by watchful waiting or TransAnal microsurgery following (chemo) Radiotherapy versus Total mesorectal excision for early REctal Cancer (STAR‐TREC study)?: protocol for a multicentre, randomised feasibility study. BMJ Open. 2017;7(12):e019474. 10.1136/bmjopen-2017-019474 PMC5770914 29288190

[51] Lee SG , Russ AJ , Casillas MA Jr . Laparoscopic transanal minimally invasive surgery (L‐TAMIS) versus robotic TAMIS (R‐TAMIS): short‐term outcomes and costs of a comparative study. Surg Endosc. 2019;33(6):1981–7. 30547391 10.1007/s00464-018-6502-8

[52] Mason S , Manoli E , Poynter L , Alexander J , Paizs P , Adebesin A , et al. Mass spectrometry transanal minimally invasive surgery (MS‐TAMIS) to promote organ preservation in rectal cancer. Surg Endosc. 2020;34(8):3618–25. 31617102 10.1007/s00464-019-07140-y PMC7326832

[53] Khokhar HA , Loughman E , Khogali M , Mulligan N , O'Shea DF , Cahill RA . Visual probing of rectal neoplasia: near‐infrared interrogation of primary tumors and secondary lymph nodes. Minerva Chir. 2018;73(2):217–26. 29471618 10.23736/S0026-4733.18.07642-3

